# Pediatric routine vaccinations in the COVID 19 lockdown period: the survey of the Italian Pediatric Society

**DOI:** 10.1186/s13052-021-01023-6

**Published:** 2021-03-24

**Authors:** Rocco Russo, Elena Bozzola, Paolo Palma, Giovanni Corsello, Alberto Villani

**Affiliations:** 1 Maternity and Pediatrics Services, Local Health Units, Benevento - The Italian Pediatric Society, Rome, Italy; 2 Pediatric and Infectious Diseases Unit, Bambino Gesù Children’s Hospital, IRCCS, Rome-The Italian Pediatric Society, Rome, Italy; 3 Clinical Immunology and Vaccinology Unit, Bambino Gesù Children’s Hospital, IRCCS, Piazza Sant Onofrio 4, 00165 Rome, Italy; 4 Department of Systems Medicine, University of Rome “Tor Vergata”, Rome- The Italian Pediatric Society, Rome, Italy; 5 Department of Sciences for Health Promotion and Mother and Child Care, University of Palermo, Palermo -The Italian Pediatric Society, Rome, Italy

**Keywords:** Vaccine, COVID, Children, Immunization

## Abstract

**Background:**

COVID-19 pandemic was responsible for disrupting routine immunization activities all over the world. Aim of the study was to investigate the reduced adherence to the national children vaccination schedule during the lockdown period in Italy.

**Materials and methods:**

Through social channels, the Italian Pediatric Society conducted a survey among Italian families on children vaccination during lockdown period.

**Results:**

In the study period, 1474 responders were collected. More than one third (34%) of them skipped the vaccine appointment as they were afraid of SARS-CoV-2-virus (44%), vaccination services postponed the appointment (42%) or was closed to public (13%).

**Discussion:**

Reduction in routine immunization coverage may represent a serious life-threating problem for unvaccinated or under-vaccinated children.

**Conclusions:**

Information on national and local preventive measures including physical distancing, handwashing, and proper coughing/sneezing hygiene should be spread among families in order to contrast vaccine hesitancy and maintain adequate coverage levels during COVID19 pandemic period.

## Background

The COVID-19 pandemic (caused by the new SARS-CoV-2 virus) risks disrupting routine immunization activities all over the world [1]. This is due to both healthcare resources being shifted to the pandemic response and the social distancing measures currently in place, as some people could decide to postpone scheduled vaccinations for themselves or their children. Public health response measures to mitigate the pandemic COVID 19 have centered on social distancing and on quarantine policies, including shelter-in-place and stay-at-home orders. During the lockdown period, children have fallen behind schedule for critical immunizations due to decreased accessibility to routine immunization services, leaving children at risk for vaccine-preventable diseases and their complications. According to data collected by the WHO, UNICEF, Gavi and the Sabin Vaccine Institute, prevision of routine immunization services is substantially hindered in at least 68 countries and is likely to affect approximately 80 million children under the age of 1 living in these countries [1]. Many countries have temporarily suspended preventive mass vaccination campaigns against diseases like cholera, measles, meningitis, polio, tetanus, typhoid, and yellow fever, due to risk of transmission of SARS-CoV-2 and the need to maintain physical distancing during the early stages of the COVID-19 pandemic. Measles and polio vaccination campaigns, in particular, have been badly hit, with measles campaigns suspended in 27 countries and polio campaigns put on hold in 38 countries. The WHO/Europe published the document “Guidance on routine immunization services during COVID-19 pandemic in the WHO European Region”. This document was designed to help countries to make decisions regarding the continuing provision of routine immunization services during the pandemic because any disruption of immunization services, even for short periods, may result in an accumulation of susceptible individuals, and a higher likelihood of vaccine preventable diseases (VPD) outbreaks [2].

The Italian Pediatric Society (SIP) investigated the decline in children vaccination during the lockdown period in Italy in order to assess the reasons why why children were not fully immunized”.

## Materials and methods

The SIP conducted a survey to Italian families through social channels and in collaboration with a digital platform, www.pazienti.it. The study period ranged from 28th April 2020 to 8th June 2020. The questionnaire was available on the digital platform of pazienti.it, on SIP website as well as on the social network pages (Twitter, Facebook, Linkedin and Telegram). The aim of the questionnaire has been explained to partecipants. The link to the questionnaire was https://docs.google.com/forms/d/e/1FAIpQLSd-Yys_bYjIVwlRIx5CyQiyPlocb8zYe45GC8XbfKfmqc_7Xw/viewform?fbclid=IwAR21zdXgbf7Nk9MOBttVZ0Wag9wJ_OXJuPIReJRQarI7nSS1XSu8B0jM5_c The unique inclusion criteria was having a child aged 0–11 years, for whom a vaccine administration was planned in the study period, according to Italian Immunization Calendar.

## Results

In the study period, 1474 answers were collected. There was not a significant difference from the geographical provenience of the answers: 658 (45%) from North of Italy (Piedmont, Aosta Valley, Liguria, Lombardy, Trentino South Tyrol, Veneto, Friuli-Venezia Giulia, Emilia-Romagna), 352 (24%) from Center (Tuscany, Umbria, Marche, Lazio) and 464 (31%) from South (Abruzzo, Molise, Campania, Apulia, Basilicata, Calabria).

As for the first questions, families were asked if they have decided to immunize their children during the lockdown. More than one third (34%) of them, skipped the vaccine appointment. The reasons for disrupted services vary. Families referred that they had their children not immunized because vaccination services postponed the appointment (42,5%) or was closed to public (13,5%). Some parents (44%) were reluctant to leave home because of restrictions on movement, lack of information, such as availability of booking child’s vaccination appointment and awareness of preventive adopted measures, or because they fear infection with the COVID-19 virus. Table 1 summarizes the results, according to geographical provenience of the answers. Even if COVID-19 pandemic emergency involved North Italian regions more than South ones, the proportion of parents who missed children’s immunization appointment during lockdown was slightly higher in the South (40% versus 34% in the North and 26% in the Center). The reasons for missing vaccine appointments were investigated. In 46% of cases, families declared not to have received enough information on national and local preventive measures including physical distancing, handwashing, and proper coughing/sneezing hygiene. As a consequence, they felt overwhelmed with worries concerning immunization in the lockdown period.

**Table 1:** Missing vaccination in children from 28th April 2020 to 8th June 2020

	Missed vaccination	Vaccination	Missed vaccination (%)	Vaccination (%)
North of Italy	435	223	66,11	33,89
Center of Italy	259	93	73,58	26,42
South of Italy	278	186	59,91	40,09

**Fig. 1:**
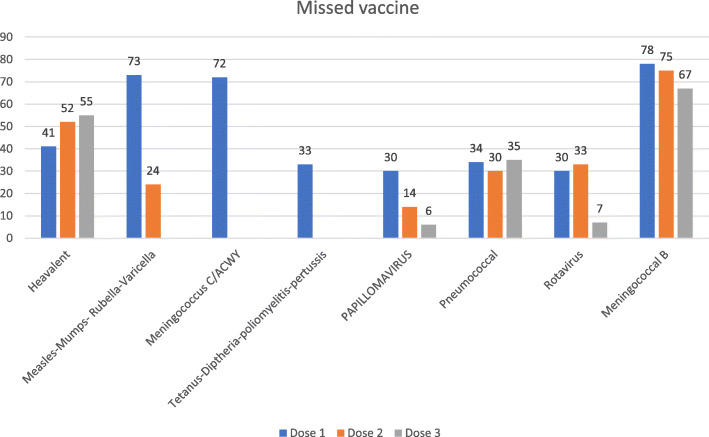
Vaccine missed in children from 28th April 2020 to 8th June 2020

Analyzing primary vaccination, Fig. 1 shows the number of skipped dose of vaccine in children in the study period. Primary vaccination appointments were missed, with the risk of re-emergence of preventable infectious diseases due to the lack of coverage.

## Discussion

In Italy, from 2017, since the introduction of compulsory vaccination, the immunization coverage progressively increased, with encouraging results [3]. Nevertheless, in the latest months, a significant drop in well-child visits has resulted in delays in vaccinations. Reduction in routine immunization coverage may represent a serious life-threating problem for un-vaccinated or under-vaccinated. The pandemic is a stark reminder of how fast an outbreak can spread without a vaccine to protect us. Disseminating accurate information and stopping the spread of misinformation is important to ensure that every child is protected with life-saving vaccines as well as seek immunization for children in case of missed appointments. In any moment and in any occasion it is important to reinforce the value of vaccination in order to prevent vaccine preventable diseases.

It is important to educate parents about measures implemented to lower the risk of acquiring COVID-19 in order to reduce fear of bringing their children into clinic for vaccines. In fact 44% of parents missed their children vaccination just for not having being well informed on the availability and safety of immunization activity. Italian regions act in different way regarding immunization services during COVID 19 lockdown, deciding whether guarantee the service, postpone some appointments or closing to public. Therefore, improve clinic access by re-opening clinics that have been closed in order to increase opportunities to vaccinate children should be listed among the strategies. Finally, provide a safe environment in which to administer vaccines may encourage families to access to vaccination.

Sanitation and hygiene services are an essential part of preventing and protecting people during infection outbreaks, such as the one caused by COVID 19. Italian vaccination centers should have modern and flexible infrastructures as well as qualified health personals in order to increase people trust in vaccination and face unpreventable outbreaks. A large-scale public awareness campaign, promoting safe vaccinations based on good handwashing behavior, hygiene, physical distancing, and the use of face masks can be of help to invert current tendency.

Discovering the reasons of childhood vaccination disrupting in Italy during the first way of COVID 19 pandemic is the strength of the survey. As the questionnaire is anonymous, we can consider the results reliable and trustable. In fact, studies suggest that when participants on survey respond anonymously instead of confidentially, the disclosure of sensitive information is enhanced [4]. Moreover, in the pandemic period an on-line survey was the best way to quickly collect information by families in order to provide corrective measures and minimize the decline in immunization practice.

The obtained results have a high implication for the control of other infectious diseases and preventable illnesses. In fact, our study confirmed that in Italy as well as in other Countries, COVID 19 caused health service disrupting and missed vaccinations among children. Moreover, the survey results may be useful in case of a future health emergency, as a “teaching lesson”. In fact, they highlight the importance of enforcing communication strategies to deliver health information. As a matter of fact, concerns about the risk of infection through engaging with other people in the absence of enough information on national and local preventive measures was one of the major cause of missed vaccination in the pediatric population.

Limitations of the study regard the control on the qualification, appropriateness and truthfulness of responders.

## Conclusion

Our study highlights that children vaccination may be missed during pandemic, mainly due to parents’ decision. Public awareness campaigns on the safety and on the importance of immunization are required in order to avoid the re-emergence of preventable infectious diseases.

Misinformation or lack of information may affect vaccination coverage, representing a risk for re-emerge of preventable infectious diseases. On the contrary, implementing and increasing opportunities and strategies to vaccinate children should be encouraged in any moment. The results may be useful to prevent immunization disrupting in case of other health emergency.

## Data Availability

At dr Russo’s repository

## Abbreviations

VPD: Vaccine preventable diseases
SIP: Italian Pediatric Society
